# Aging effects of the female pelvis and organ endo-pelvic fascia space as shown by novel MRI measurements

**DOI:** 10.1097/MD.0000000000045922

**Published:** 2026-01-09

**Authors:** Yun Chen, Cheng Qian, Yan Yin, Yinjian Zhou, Zhuangzhuang Xu, Xiangming Fang

**Affiliations:** a Department of Obstetrics and Gynecology, Huzhou Maternity and Child Health Care Hospital, Huzhou, Zhejiang, China; b Department of Surgery, Huzhou Maternity and Child Health Care Hospital, Huzhou, Zhejiang, China; c Department of Imaging, Huzhou Maternity and Child Health Care Hospital, Huzhou, Zhejiang, China.

**Keywords:** age, magnetic resonance imaging, pelvic floor anatomical structure, pelvic floor dysfunction, pelvis

## Abstract

This study investigated age-related anatomical changes in female pelvic structures using magnetic resonance imaging (MRI), aiming to identify factors contributing to pelvic floor dysfunction. This was a retrospective cross-sectional study including pelvic MRI data from 120 asymptomatic women (aged 15–74 years), divided into 6 age groups at 10-year intervals. Measured parameters included pubic symphysis inclination angle, anterior bladder space area, puborectal muscle pinch angle, and rectovaginal-anal levator gap area. All parameters demonstrated significant age-related trends. The pubic symphysis inclination angle increased progressively, reaching its highest mean value in women aged 65 to 74 years (51.8° vs 41.2° in the 15–24 group, *P* < .05). The anterior bladder space area was significantly larger in women ≥45 years (mean 7.9 cm²) compared with those <45 years (mean 5.1 cm², *P *< .05). The puborectal muscle pinch angle was greatest in the youngest group (mean 91.3° in 15–24 vs 83.7° in older groups, *P *< .05). The rectovaginal-anal levator gap area expanded notably in the 65 to 74 group (2.3 cm² vs 1.5 cm² in younger groups, *P* < .05). Correlation analysis confirmed positive associations between all parameters and advancing age. Progressive age-related changes in pelvic anatomy and musculature may predispose women to pelvic floor dysfunction, particularly pelvic organ prolapse. Quantitative MRI provides objective markers for early detection of pelvic floor weakening and may serve as a valuable tool for screening and monitoring in middle-aged and elderly parous women to guide timely preventive interventions.

## 1. Introduction

The occurrence of female pelvic floor disorders is a complex problem that can be recognized anatomically by some classical theories. Since 1908, Fothergill and DeLancey^[1-3]^ have proposed the holistic theory of the pelvic floor, the three-level theory and the hammock hypothesis, suggesting that the structure and function of the female pelvic floor is an interconnected and dynamic anatomical whole. While women go through pregnancy phase, during which there are significant changes in pelvic floor morphology, some of which occur after delivery.^[4]^ Remodeling during pregnancy and/or injury during vaginal delivery can have lasting effects on the pelvic floor muscles, fascia, ligaments and other tissues.^[5]^ In addition, several studies have shown that age-related changes are an independent risk factor for the decline of pelvic floor support.^[6]^ Therefore, further study of the female pelvic floor anatomy is an essential pathway to explore the pathogenesis of female pelvic floor dysfunction (PFD) diseases.

Pelvic floor magnetic resonance imaging (MRI) is recognized as the most preferred method for comprehensive assessment of pelvic floor anatomy and defects because of its high soft tissue resolution.^[7]^ On pelvic floor MRI images, the pelvic floor is divided into 3 main layers from the outside to the inside, with the outer layer referring to the superficial fascia and superficial muscles, the middle layer referring to the perineal diaphragm (also known as the genitourinary diaphragm), and the inner layer being the pelvic diaphragm. The connective tissues of the pelvis, perineal diaphragm and intrapelvic fascia provide passive support to the pelvic floor, while the pelvic diaphragm layer provides active support to the pelvic floor through timely contraction and relaxation.^[8]^ Cheng et al^[9]^ studies showed that MRI images suggest some changes in the pelvis and muscles of women with increasing age, and can be used as a diagnostic basis for women with and without prolapse.^[10]^ However, there is still a lack of systematic research on how pelvic bones, muscles, and fascia change across different age groups in women. To fill this gap, the present study represents the first application of a multi-index MRI parameter system to systematically evaluate the pelvic floor structure of women across different age groups. By simultaneously assessing the pubic symphysis inclination angle, anterior bladder space area, puborectal muscle pinch angle, and rectovaginal-anal levator gap area, our study provides a comprehensive and quantitative framework for characterizing age-related pelvic floor changes. This innovative approach not only delineates continuous anatomical trends with aging but also offers imaging-based evidence that may guide early screening, monitoring, and preventive interventions for PFD.

## 2. Materials and methods

This was a retrospective study using survey data at Huzhou Maternity & Child Health Care Hospital, and 120 female patients who met the following criteria and received medical care and examinations at the hospital between 2020 and 2023 were included in the study. Ethical approval for this study was obtained from the Ethics Committee of Huzhou Maternity & Child Health Care Hospital (Ethical Approval No. 2023-J-059). Every patient was approached and only participants who provided informed consent were enrolled for the study. Women who declined to provide informed consent were excluded from the study. The inclusion criteria were: age between 15 and 74 years; no pelvic surgery other than cesarean delivery; no definite PFD diseases; no pelvic floor tumors or malformations causing axial changes in the pelvic floor; and no contraindications to other MRI examinations such as claustrophobia and pelvic metal implants. The patients were divided into 6 age groups of 10 years: 15 to 24 years, 25 to 34 years, 35 to 44 years, 45 to 54 years, 55 to 64 years, and 65 to 74 years, and all patients underwent static pelvic floor MRI scans. Prior to the scans, they were informed that their examination data might be used for medical research analysis in the future and signed informed consent forms. Patients meeting the aforementioned criteria, grouped by age, were then included in this retrospective study. The basic information of the groups is shown in Table 1.

**Table 1:** Baseline characteristics of participants by age group.

Characteristic	15–24 yr (n = 20)	25–34 yr (n = 20)	35–44 yr (n = 20)	45–54 yr (n = 20)	55–64 yr (n = 20)	65–74 yr (n = 20)	Total (n = 120)	*P*-value
Age, mean ± SD (yr)	20.1 ± 2.5	29.7 ± 3.1	39.6 ± 2.9	49.8 ± 3.2	59.7 ± 2.7	69.4 ± 3.0	44.9 ± 15.7	–
BMI, mean ± SD (kg/m²)	21.3 ± 2.1	22.1 ± 2.3	22.8 ± 2.6	23.4 ± 2.5	23.9 ± 2.8	24.2 ± 2.7	23.0 ± 2.6	.08
Parity, n (%)	0 (0%)	5 (25%)	12 (60%)	16 (80%)	18 (90%)	20 (100%)	71 (59.2%)	< .001
Cesarean delivery, n (%)	0	2 (10%)	4 (20%)	3 (15%)	2 (10%)	1 (5%)	12 (10%)	.27
Vaginal delivery, n (%)	0	3 (15%)	8 (40%)	13 (65%)	16 (80%)	19 (95%)	59 (49.2%)	< .001
Menopausal status, n (%)	0	0	2 (10%)	8 (40%)	18 (90%)	20 (100%)	48 (40%)	< .001
Hormone therapy, n (%)	0	0	1 (5%)	2 (10%)	3 (15%)	2 (10%)	8 (6.7%)	.42

SD = standard deviation.

## 3. Methods

### 3.1. MRI scan

All subjects underwent pelvic MRI using a 1.5-T MAGNETOM Avanto scanner with a surface body coil (Siemens Healthineers AG, Erlangen, Germany). Patients were instructed to moderately fill the bladder before scanning and remained in the supine position with legs extended during the examination. Static pelvic floor images were obtained in sagittal, transverse, and coronal planes with T1-weighted imaging and T2-weighted imaging sequences. Each sequence was acquired in 10 layers. Images were reviewed by 2 senior radiologists, and the areas of interest were manually outlined on the picture archiving and communication system workstation to automatically generate measurement data.

Detailed MRI acquisition parameters (repetition time/echo time, field of view, matrix size, slice thickness, etc) are provided in Table 2.

**Table 2:** Detailed MRI acquisition parameters.

Sequence	TR/TE (ms)	FOV (mm)	Matrix	Slice thickness (mm)	Other parameters
Sagittal T2WI_SSTSE	4000/79	250 × 250	320 × 320	5	Nex = 1
Sagittal T1WI_SSTSE	520/11	250 × 250	320 × 320	3	Nex = 2
Transverse T2WI_SSTSE	4000/79	350 × 350	512 × 512	3	Nex = 2, echo train = 256, flip angle = 180°
Transverse T1WI_SSTSE	570/12	350 × 350	512 × 512	3	echo train = 256, flip angle = 180°
Coronal T2WI_SSTSE	4170/101	320 × 320	320 × 320	5	Nex = 2

FOV = field of view, MRI = magnetic resonance imaging, T1WI = T1-weighted imaging, T2WI = T2-weighted imaging, TE = echo time, TR = repetition time.

### 3.2. Image processing and analysis

**Figure 1.**
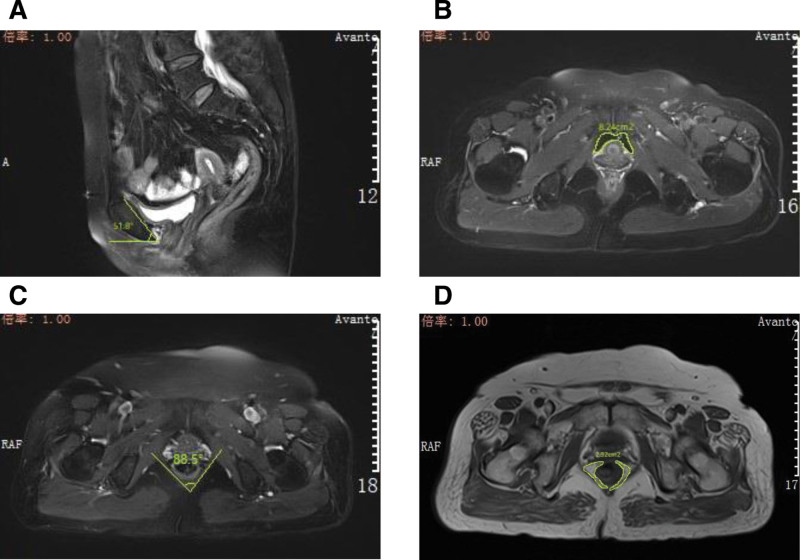
MRI parameters. (A) Pelvic floor MRI, sagittal T1WI sequence image of a 68-year-old woman with a pubic symphysis inclination angle of 51.8°. (B) Pelvic floor MRI with transverse T1WI sequence images of a 66-year-old female with an anterior bladder space area of 8.24 cm^2^. (C) Pelvic floor MRI with a transverse T1WI sequence image of a 67-year-old female with a puborectal muscle pinch angle of 88.5°. (D) Pelvic floor MRI with a transverse compression lipid T2WI sequence image of a 67-year-old female with a rectovaginal-anal levator gap area of 2.32 cm^2^. MRI = magnetic resonance imaging, T1WI = T1-weighted imaging, T2WI = T2-weighted imaging.

The following parameters were measured on the picture archiving and communication system workstation by 2 radiologists (with 10 years’ experience in reading pelvic MR films). The final values were taken as the mean of the two measurements. MRI parameters – the pubic symphysis inclination angle: the angle between the pubic symphysis and the horizontal line was measured in the median sagittal plane (Fig. 1A); the anterior bladder space area: the area of the posterior bladder space was calculated by taking the midpoint of the pubic symphysis in the transverse position (Fig. 1B); the puborectal muscle pinch angle: take the angle of the puborectal muscle bilaterally in the transection position (Fig. 1C); the rectovaginal-anal levator gap area: in a transverse position, the area enclosed by the anterolateral rectal wall, the posterior vaginal wall and the bilateral anal levator muscle (Fig. 1D).

### 3.3. Statistical analysis

Using SPSS 23 statistical analysis software, all measurements were expressed in square centimeters (cm^2^) for area and in degrees (°) for angle, to 2 decimal places. The measures were statistically described as mean ± standard deviation ($\bar{x}\pm s$). One-way analysis of variance was used to compare the parameters among the 6 groups,and independent sample *t*-test was used to compare the parameters of each age group separately. All statistical analyses were performed at *P* < .05 as statistically significant differences. Spearman correlation analysis was used to assess whether the pelvic floor MRI parameters were correlated with age in each group.

## 4. Results

### 4.1. Comparison of pelvic floor MRI parameters between the 6 groups

The statistical data of pelvic floor MRI parameters among the 6 groups are shown in Table 3. The differences in symphysis pubis inclination angle, anterior bladder space area, puborectal muscle angle, and rectovaginal-anal levator gap area among the 6 groups were statistically significant (*P *< .05). Pairwise comparison within groups showed that: pubic symphysis inclination angle: the difference between the 65 to 74 years and the other 5 groups was statistically significant (*P* < .05); anterior bladder space are the differences among the age groups of 15 to 4 years, 25 to 34 years, and 35 to 44 years were not statistically significant (*P* > .05). Similarly, the differences among the age groups of 45 to 54 years, 55 to 64 years, and 65 to 74 years were not statistically significant (*P *> .05). However, the differences between these 2 major groups were statistically significant (*P* < .05), indicating a significant change in this area among women around the age of 45; puborectal muscle pinch thee: the difference between the 15 to 24 years and the other 5 groups was statistically significant (*P* < .05); rectovaginal-anal levator gap area: the difference between the 65 to 74 years and the other 5 groups was statistically significant (*P* < .05).

**Table 3:** Comparison of measurements between age groups ().

Group	Age group	Pubic symphysis inclination angle (°)	Anterior bladder space area (cm^2^)	Puborectal muscle pinch angle (°)	Rectovaginal-anal levator gap area (cm^2^)
Group 1	15–24 yr old	34.84 ± 1.57	3.09 ± 0.17	46.29 ± 1.62*	0
Group 2	25–34 yr old	35.12 ± 1.53	3.49 ± 0.24	59.46 ± 3.03	0
Group 3	35–44 yr old	35.98 ± 1.17	3.53 ± 0.21	55.22 ± 2.40	0
Group 4	45–54 yr old	37.74 ± 1.51	4.48 ± 0.37†	57.93 ± 3.09	0
Group 5	55–64 yr old	38.37 ± 1.41	5.01 ± 0.37†	56.55 ± 3.92	0.28 ± 0.24
Group 6	65–74 yr old	42.96 ± 1.87*	5.29 ± 0.41†	59.46 ± 3.70	0.47 ± 0.17*
	*P*	.002	.000	.028	.022

*P* < .05.

* indicates a statistically significant difference when compared with other subgroups.

† indicates a statistically significant difference compared to groups 1–3.

### 4.2. Correlation analysis of pelvic floor MRI parameters with age

**Figure 2.**
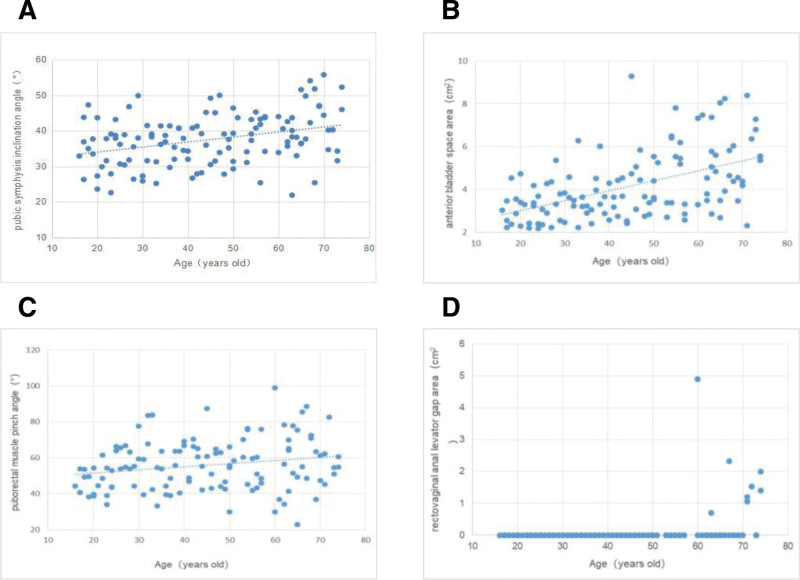
Correlation analysis of pelvic floor MRI parameters with age. (A) Pubic symphysis inclination angle was positively correlated with age (*r* = 0.320, *P* *<* .0001). (B) Anterior bladder space area was positively correlated with age (*r* = 0.503, *P* < .0001). (C) Puborectal muscle pinch angle was positively correlated with age (*r* = 0.220, *P* = .016). (D) Rectovaginal anal levator gap area was positively correlated with age (*r* = 0.378, *P* *<* .0001). MRI = magnetic resonance imaging.

Pubic symphysis inclination angle (Fig. 2A, *r* = 0.320, *P* < .0001), anterior bladder space area (Fig. 2B, *r* = 0.503, *P *< .0001), puborectal muscle pinch angle (Fig. 2C, *r* = 0.220, *P* = .016), and rectovaginal-anal levator gap area (Fig. 2D, *r* = 0.378, *P *< .0001) were all positively correlated with age, see Figure 2.

## 5. Discussion

Pubic symphysis inclination angle: the pelvis is the most gender-specific part of the human skeleton, which unites the iliac and pubic bones on both sides through the pubic symphysis. For women, both morphologically and functionally, the pelvis is adapted to the obstetric requirements of bipedal walking and safe delivery.^[11]^ The angle of the pubic symphysis indirectly reflects the tilt angle of the pelvis, and changes in the tilt angle of the pelvis also have an effect on the pelvic floor load bearing. Cinzia Fornai et al^[12]^ have shown that in adolescence, the superior margin of the pubic symphysis is skewed forward, and with age, the pelvis is rearranged, with the ilium more vertical and the superior pubic branch more backward near the symphysis. During the perinatal period to facilitate the infant’s head into the pelvis, access to the birth canal, and rotation, the ligaments connecting the pubic bones must stretch and the pubic symphysis separate, so the inclination of the pubic symphysis changes at the same time.^[13]^ It can be concluded from our data that this angle of inclination increases linearly with age, with an average difference in angle of nearly 10° between the 15 to 24 and 65 to 74 age groups. In younger women the bladder and other organs are equivalent to lying on the pubic symphysis, while in older women the bladder is equivalent to half hanging on the pubic symphysis. In women, the angle of inclination of the pubic symphysis gradually increases from young to old, and the fattening of the triangular ligament and the pubourethral and pubocervical ligaments causes a decrease in adhesion, similar to a “slippery slope.” Therefore, an increase in the tilt of the pubic symphysis also means an overall posterior tilt of the pelvis, with the resulting horizontal pelvic floor plane and increased vertical pressure.

Anterior bladder space area: The anterior bladder space behind the pubic bone is formed by thickening of the pelvic wall fascia below the midpoint of the pubic symphysis, named the triangular ligament of the bladder and the pubourethral ligament, which serves to suspend the urethra and the bladder neck; in addition, the anterior space contains fat, loose fibrous tissue and venous plexus.^[13,14]^ The data in the this paper indicate that differences in the area of the anterior bladder space occur with increasing age. Taking 45 years old as the boundary, the age group younger than 45 years is obviously smaller than the age group older than 45 years. This may be related to the beginning of perimenopause in women around the age of 45 years, when estrogen levels decline, leading to muscle and ligament laxity and steatosis in the pelvis.^[15]^ It can be concluded from our data that the area gradually increases with age, which also suggests the area of the anterior bladder space as a possible participating factor in the occurrence of pelvic organ prolapse.

Puborectal muscle pinch angle: in ultrasonography, the area of the anal raphe fissure is measured as a basis for determining the degree of prolapse.^[16,17]^ In this study, we used instead a simple method of MRI measurement of the puborectalis muscle pinch angle for assessment. The data in the paper showed that the puborectalis angle widened in the age group after 25 years and did not continue to widen with increasing age thereafter. It is suggested that the angle of the puborectalis muscle increased significantly in women after pregnancy compared to women without pregnancy, that women who experienced pregnancy and childbirth did not return to their prepregnancy state after delivery, and that the angle did not continue to expand thereafter. Therefore, the effect of pregnancy and childbirth on the female pelvic floor muscles is lifelong,^[18]^ the perinatal period makes the female pelvic floor muscle extension can be said to be to the peak, and thereafter there is no chance to fully return to prepregnancy on its own, which may also be related to the expansion of the pelvis, which provides a channel for pelvic organ prolapse and becomes the basis for the pathogenesis of PFD diseases.^[19]^

Rectovaginal-anal levator gap area: we know that in men the intrinsic rectal fascia fuses with the surface fascia of the anal raphe and the Denonvilliers fascia anteriorly to form a slightly thickened white fascial structure^[20]^ and thickens directly posteriorly to form the hiatal ligament to fix end of the rectum.^[21]^ Tsukada et al^[22]^ found that the fibers of the anal raphe inserted longitudinally into the rectal wall play an important role in the fixation of the rectum The anorectalis muscle does not have muscle fibers inserted into the vagina, only tightly bonded by fascia. Meanwhile, Zhang et al^[23]^ found that the female rectovaginal septum is not a fascial structure, but is filled with adipose tissue, loose reticular fibrous tissue and some muscle fibers. These septa pull on each other and play an important role in maintaining the pelvic organs, increasing adhesion, and limiting the spread of inflammatory or malignant tumors.^[24]^ Therefore, in anatomical studies of pelvic floor structures, it is considered that the rectovaginal septum and the lateral rectal fascial mesh pocket-like support are significant for the stability of the posterior pelvis. Weakness in this area is the main cause of posterior pelvic organ prolapse and rectal prolapse. Rodríguez-Abarca et al^[25]^ found that the rectovaginal septum is significantly longer in premenopausal women and decreases in thickness after menopause. Our data suggest that with increasing age, degeneration of the fascias between the rectovaginal-anal septum is seen and eventually the attachment weakens, and in a few patients, a significant gap even begins to appear, with the consequent weakening of the support of the pelvic floor muscles to the posterior vaginal wall, causing prolapse.

Further refine and establish quantitative evaluation methods for female pelvic floor MRI, particularly focusing on timely monitoring and early warning of pelvic floor structure in middle-aged and elderly women who have given birth, to prevent pelvic organ prolapse. When assessing pelvic floor function, attention should be paid to changes in pelvic bone structure, adhesions between pelvic organs, and the comprehensive consideration of these factors. Note that pelvic floor structure and morphology may change with increasing age, which can lead to greater stability. This study did not differentiate between groups with multiple pregnancies or multiple deliveries, nor did it distinguish between different delivery methods such as vaginal delivery, difficult vaginal delivery, and cesarean section, or measure differences in various indicators among each age group. Future research could refine this aspect of the work.

This study has several limitations. First, it was conducted in a single center with a relatively limited sample size, which may restrict the generalizability of the findings. Second, the retrospective cross-sectional design only allows us to identify associations between age and pelvic floor structural changes, but it cannot establish causal relationships. Third, no symptom-related clinical data (such as urinary incontinence, pelvic organ prolapse stage, or quality-of-life scores) were collected, which limits the ability to directly link MRI changes to functional outcomes. In addition, we did not differentiate between women with different numbers or methods of delivery, which may be important confounding factors influencing pelvic floor structures.

Despite these limitations, our findings have potential clinical implications. Quantitative MRI evaluation of the pelvic floor could be particularly useful for early detection of pelvic floor weakening in middle-aged women (≥45 years), postmenopausal women, and multiparous women, who are at higher risk of PFD. Integrating MRI-based assessment into routine gynecological or imaging examinations could help identify patients requiring closer follow-up, guide preventive strategies such as pelvic floor muscle training, and provide objective parameters for preoperative evaluation in women considering pelvic reconstructive surgery.

## Author contributions

**Conceptualization:** Yun Chen, Cheng Qian, Xiangming Fang.

**Data curation:** Cheng Qian, Xiangming Fang.

**Formal analysis:** Yun Chen, Cheng Qian.

**Investigation:** Yun Chen, Zhuangzhuang Xu.

**Methodology:** Yun Chen, Cheng Qian, Yan Yin.

**Supervision:** Yun Chen, Cheng Qian, Yinjian Zhou, Xiangming Fang.

**Validation:** Yinjian Zhou, Zhuangzhuang Xu, Xiangming Fang.

**Visualization:**Yan Yin, Xiangming Fang.

**Writing – original draft:** Yun Chen, Cheng Qian, Xiangming Fang.

**Writing – review & editing:** Yun Chen, Cheng Qian, Xiangming Fang.
